# Empirical Data Suggest That the Kashmir Musk Deer (*Moschus cupreus*, Grubb 1982) Is the One Musk Deer Distributed in the Western Himalayas: An Integration of Ecology, Genetics and Geospatial Modelling Approaches

**DOI:** 10.3390/biology12060786

**Published:** 2023-05-29

**Authors:** Amira Sharief, Bheem Dutt Joshi, Vineet Kumar, Hemant Singh, Vinay Kumar Singh, Shahid Ahmad Dar, Catherine Graham, Chinnasamy Ramesh, Iyaz Quyoom, Mukesh Thakur, Lalit Kumar Sharma

**Affiliations:** 1 Zoological Survey of India, Kolkata 700053, India; 2 Wildlife Institute of India, Dehradun 248001, India; 3 WSL Swiss Federal Research Institute, 8903 Zurcherstrasse, Switzerland; 4 Department of Zoology, University of Kashmir, Srinagar 19006, India

**Keywords:** Kashmir musk deer, Western Himalayas, endangered, DNA, camera trapping, species distribution modelling

## Abstract

**Simple Summary:**

Resolving the taxonomic ambiguity of a species is important for their long-term conservation. We used non-invasive DNA sampling and camera trapping to address the taxonomic ambiguity in musk deer species in the Western Himalayas, and species occurrence locations were used to model the species distribution to identify suitable habitats. The combined results confirm the presence of only Kashmir musk deer (KMD) in the Western Himalayas. During our survey, no other musk deer species were found in the study area. This finding suggests that the presence of other species, such as Himalayan musk deer and Alpine musk deer, were incorrectly reported in the past. The predicted distribution (6% of the total area) of Kashmir musk deer is a narrow belt of the Western Himalayas between 2500 and 4500 m. Based on this study, we recommend long-term monitoring and assessment of KMD throughout its distribution range for its conservation and management.

**Abstract:**

Insufficient research has been conducted on musk deer species across their distribution range, primarily because of their elusive behaviour and the fact they occupy remote high-altitude habitats in the Himalayas above 2500 m. The available distribution records, primarily derived from ecological studies with limited photographic and indirect evidence, fail to provide comprehensive information on the species distribution. Consequently, uncertainties arise when attempting to determine the presence of specific taxonomic units of musk deer in the Western Himalayas. This lack of knowledge hampers species-oriented conservation efforts, as there need to be more species-specific initiatives focused on monitoring, protecting, and combatting the illegal poaching of musk deer for their valuable musk pods. We used transect surveys (220 trails), camera traps (255 cameras), non-invasive DNA sampling (40 samples), and geospatial modelling (279 occurrence records) to resolve the taxonomic ambiguity, and identify the suitable habitat of musk deer (*Moschus* spp.) in Uttarkashi District of Uttarakhand and the Lahaul–Pangi landscape of Himachal Pradesh. All the captured images and DNA-based identification results confirmed the presence of only Kashmir musk deer (KDM) (*Moschus cupreus*) in Uttarakhand and Himachal Pradesh. The results suggest that KMD inhabit a narrow range of suitable habitats (6.9%) of the entire Western Himalayas. Since all evidence indicates that only KMD are present in the Western Himalayas, we suggest that the presence of other species of musk deer (Alpine musk deer and Himalayan musk deer) was wrongly reported. Therefore, future conservation plans and management strategies must focus only on KMD in the Western Himalayas.

## 1. Introduction

Musk deer are solitary, shy, and crepuscular/nocturnal animals distributed in the continuous to fragmented patches of alpine scrublands and forested habitats of the mountain regions in 13 Asian countries [1,2,3,4]. Musk deer are the least studied species among the ungulates. However, they have a specific role in alpine scrub habitats and are considered ecological indicators for ecosystem stability [5]. A total of seven species of musk deer have been described globally, viz., Chinese forest musk deer (*M. berezovskii*), Siberian musk deer (*Moschus moschiferus*), Alpine musk deer (*M. chrysogaster*), Himalayan musk deer (*M. leucogaster*), Black musk deer (*M. fuscus*), Anhui musk deer (*M. anhuiensis*), and Kashmir musk deer (*M. cupreus*) [2,3,6,7]. While their fine-scale distribution boundaries are yet to be mapped with concrete evidence supporting their presence in the different geographic areas, out of seven species, four species (*M. cupreus*, *M. fuscus*, *M. chrysogaster*, and *M. leucogaster*) are reported to be distributed in India in fragmented patches all along the higher elevation region (2500–4500 m) of Uttarakhand, Himachal Pradesh, the Union territory of Jammu and Kashmir (Western Himalayas), Sikkim (Central Himalayas), and Arunachal Pradesh (Eastern Himalayas) in the Indian Himalayan region [8]. These species recently gained attention among biologists after a drastic population decline due to intensive poaching for musk pods [9,10,11]. Increased anthropogenic activities such as livestock grazing, fuel-wood collection, habitat fragmentation, developmental projects (road networks and river valley projects), and climate change are the other key degradation drivers responsible for the decline in its population [10,11,12,13]. Consequently, six species of musk deer are listed as ‘Endangered’ in the IUCN Red List of Threatened Species [13] Appendix-I of the ‘Convention on International Trade in Endangered Species of Wild Fauna and Flora’ (CITES) [14] and are also listed in Schedule-I of India’s Wildlife (Protection) Act (1972).

Previous studies suggest the presence of both Alpine musk deer (*M. chrysogaster*) and Himalayan musk deer (*M. leucogaster*) in Sikkim, Uttarakhand, Himachal Pradesh, and Arunachal Pradesh [3,4,15,16,17]. However, the confirmed records of the presence of these two species from the Western Himalayas are not based on DNA-based techniques or camera trap results. Moreover, Kashmir musk deer (*Moschus cupreus*, henceforth KMD) was only reported from the westernmost limits of the Hindu Kush Himalaya in Jammu and Kashmir, India, and Afghanistan [2,18]. Recent genetic evidence revealed that the musk deer species present in Uttarakhand is a distinct lineage from all other known species [4], which was subsequently confirmed as KMD in Uttarakhand and central Nepal [19,20]. However, ambiguities still prevail in musk deer about the number of species/sub-species, taxonomy, and distribution in India [3,4]. Hence, resolving the taxonomic ambiguity and uncertainty in the distribution range of musk deer species in the Western Himalayas is essential to develop a species-oriented conservation programme. Therefore, the present study aimed to understand how many species of musk deer are distributed within the possible habitat range of musk deer in the Western Himalayas.

We used different wildlife survey methods (camera trapping, a sign survey, non-invasive DNA sampling, and species distribution modelling) to resolve the taxonomy and distribution of musk deer in the Western Himalayas. Camera trapping is one of the most accepted and used non-invasive methods to detect elusive species to understand the occurrence, relative abundance, habitat choice, activity pattern, density estimation, and species inventory [21,22,23]. Furthermore, the non-invasive DNA-based approach provides essential information about a species’ occurrence, taxonomy, distribution, status, and evolutionary history [19,24,25]. The confirmed species presence location data from different methods accurately help to understand the distribution, delineate species boundaries, and identify suitable habitats using species distribution modelling [24,26,27,28]. Therefore, the present study integrates the different methods to delineate the species boundaries, assess the distribution range, and predict the suitable habitat of musk deer using mitochondrial DNA markers, camera trapping, a sign survey, and species distribution modelling in the Western Himalayas.

## 2. Material and Methods

### 2.1. Study Area

**Figure 1:**
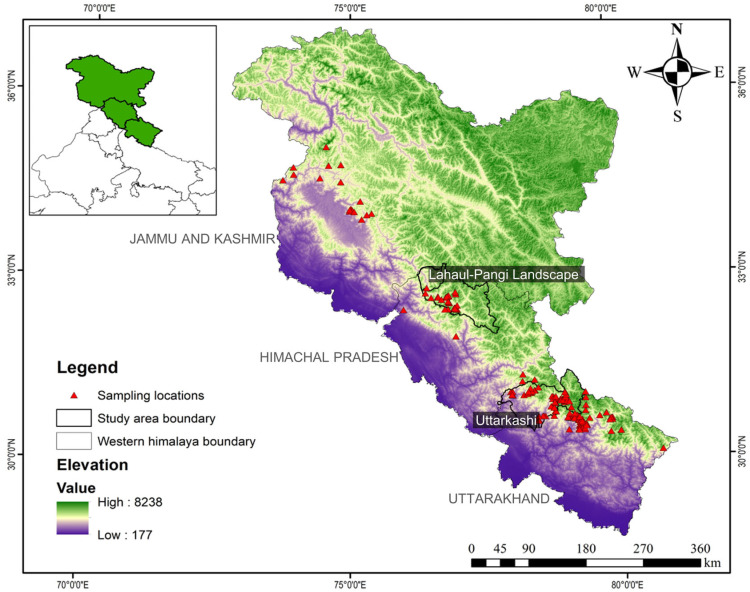
Map showing the presence locations of Kashmir musk deer in the Western Himalayas region of India.

This study was conducted in the Uttarkashi District of Uttarakhand, Lahaul Valley of Lahaul and Spiti District, and Pangi Valley of Chamba District of Himachal Pradesh in the Western Himalayan region of India (Figure 1). We divided the study landscape into 10 km × 10 km grids to conduct the reconnaissance survey. Further, based on the species presence in different elevation ranges and forest types, we intensified the study area into 2 km × 2 km grids [17,20,27]. We covered all the logistically accessible grids in the distribution range of musk deer in both landscapes. Further, for performing species distribution modelling, we used the entire Western Himalayan range of India (Figure 1).

### 2.2. Camera Trapping, Sign Survey and Non-Invasive Genetics Sampling

We placed 134 camera traps in Uttarkashi District of Uttarakhand, 105 in Lahaul Valley of Lahaul and Spiti District, and 16 in Pangi Valley of Chamba District of Himachal Pradesh (henceforth, Lahaul Valley and Pangi Valley are called Lahaul–Pangi landscape). To detect the species, we employed an ultra-compact SPYPOINT FORCE-11D trail camera (manufactured by SPYPOINT, GG Telecom, Quebec, QC, Canada) and Browning trail camera traps (specifically the Defender 850 model, with 20 MP, produced by Prometheus Group, LLC based in Birmingham, Alabama; website: https://browningtrailcameras.com). The cameras were mounted 30–45 cm above ground on natural trails, in valleys, and in the identified latrine sites of musk deer, without lures [27]. We also traversed a total of 106 trails in Lahual Valley, 99 in Uttarkashi, and 15 in Pangi Valley of, 2–6 km in length, to collect non-invasive samples. A total of 172 non-invasive faecal pellets samples (116 Lahaul Valley, 54 Uttarkashi, and 2 Pangi Valley) were collected during our survey period of two years, 2018–2020. We also identified 32 latrine sites of musk deer in the Lahaul–Pangi landscape and 11 latrine sites in Uttarkashi (based on specific characteristics) since no other overlapping species is found within that range, especially in the Lahaul–Pangi landscape [20]. In addition, we collected putative faecal pellets of musk deer origin, air-dried them in field conditions, and further stored them in 50 mL sterilised silica-containing vials for subsequent DNA analysis.

### 2.3. DNA Extraction, PCR Amplification, and Species Identification

The collected samples were processed for DNA extraction, wherein 20 samples were randomly selected from each locality. The genomic DNA was extracted using the QIAamp DNA Stool Mini Kit (Qiagen, Hilden, Germany) in accordance with the instructions of the manufacturer. All the extracted samples of musk deer were PCR-amplified using the mitochondrial cytochrome b gene of 401 bp (mcb398 ‘TACCATGAG-GACAAATATCATTCTG’ and mcb869 ‘CCTCCTAGTTTGTTAGGGATTGATCG’) designed by Verma and Singh (2003) [29]. The PCR reactions were carried out in a 10 μL PCR, containing 2.0 μL DNA template, 0.8 mM Mgcl_2_, 2× Buffer, 0.3-unit Tag DNA polymerase, 1.0 μM of each primer, and 2.0 μL dNTPs. PCR thermal cycles were set with an initial denaturation of 5 min at 94 °C, followed by 40 cycles of denaturation (45 s at 94 °C), annealing (60 s at 55 °C), and extension at (1 min at 72 °C), with a final extension of 72 °C for 10 min. We used negative control during DNA extractions and PCR amplification to identify and eliminate potential contamination. The PCR product was used for cycle sequencing, cleaned using the ‘BigDye Terminator’ sequencing kit (Applied Biosystems, Waltham, MA, USA), and subjected to DNA sequencing on the Genetic Analyzer (ABI 3730; Applied Biosystems, MA, USA).

### 2.4. Data Analysis

#### 2.4.1. Non-Invasive DNA Sampling

All the sequences were examined and processed for quality in Sequencher 4.7 (Gene Codes Corporation, Ann Arbor, MI, USA). Multiple sequence alignment (MSA) was carried out using the CLUSTAL W programme implemented in BioEdit version 7.0.90 [30], and then the alignments were verified by visually inspecting the sequences. Further, cleaned sequences were validated with reference data through the NCBI BLAST tool of GenBank (http://www.ncbi.nlm.nih.gov (accessed on: 1 February 2021)). The taxonomic units (TUs) were detected by having above 98% similarity of our sequences, with reference sequences available in NCBI (whole mitochondrial genome of Kashmir musk deer: accession numbers MT873041.1 and MT873042.1) and with intra-species sequence divergence of <2%. We used *Cervus elaphus* (Genbank accession no. NC_014703.1) as an outgroup species for reconstructing the phylogenetic tree. We then retrieved the sequences of other musk deer species from the NCBI to understand the phylogenetic relationship. Sequence divergence (using Kimura 2 parameters (K2P), neighbour-joining, and maximum likelihood phylogenetic tree were reconstructed using MEGA11 [31], while the Bayesian-based phylogenetic tree was reconstructed using Beast programme version 2.1.3 [32]. To reconstruct the phylogenetic tree, we utilised the HKY nucleotide substitution model selected by Mr Modeltest V.2 [33]. The Markov Chain Monte Carlo (MCMC) simulation was conducted for 20 million generations, with trees sampled at every 1000th generation. To ensure convergence, we discarded the initial 20% of trees, considered burn-in. The phylogenetic tree was then annotated and visualised using Tree-Annotator version 1.8.1 and FigTree version 1.3.1. [32], respectively.

#### 2.4.2. Camera Traps and Sign Survey

All the images of camera traps were sorted species-wise [34], species were identified by referring field manual [34] with expert advice, and unidentified images were discarded from the analysis. The capture rate (total number of independent captures/total trap nights) of musk deer was calculated using the identified number of camera trap images. The number of camera trap nights was computed from the camera’s deployment to its retrieval. We only evaluated a second capture after a one-hour break for large mammals [27,35,36]. The encounter rate (ER; total number of signs/trails walked in km) was also calculated using indirect/direct evidence recorded during the sign survey.

### 2.5. Species Distribution Modelling

#### 2.5.1. Occurrence Data

Occurrence data of musk deer were collected during 2018–2020 in three landscapes of the Western Himalayas, viz., Uttarkashi, Lahaul Valley, and Pangi Valley. We obtained 279 occurrence records of musk deer through primary surveys and from the published studies from the western Himalayan region of India [4,17,18,19,37]. Out of the total, only 220 spatially independent occurrence records of KMD were used for modelling the distribution in the western Himalayan region of India (Figure 1). The spatial autocorrelation of the locations was tested using the ‘SDM toolbox’ in ArcGIS 10.9.

#### 2.5.2. Data Preparation

We used four categories of environmental variables (bioclimatic, topographic, LULC, and anthropogenic variables) for modelling the distribution of KMD. The bioclimatic variables are represented by 19 variables extracted from World Clim Ver. 2 (www.worldclim.org (accessed on 1 March 2022)) with ~1 km resolution [38]. The land use land cover (LULC) type was retrieved from the Moderate Resolution Imaging Spectroradiometer (MODIS) and Land Cover Type Product (MCD12Q1) with a 500-m resolution and was classified into 17 different LULC classes https://earthexplorer.usgs.gov/ (accessed on 1 March 2022). We used the Shuttle Radar Topographic Mission (SRTM) image downloaded from Earth Explorer (https://earthexplorer.usgs.gov/ (accessed on 1 March 2022)) to prepare the topographic variables such as elevation, slope, and aspect. KMDs are sensitive to anthropogenic disturbances; hence, we also used the global human footprint dataset downloaded from the Socioeconomic Data and Applications Centre SEDAC, NASA (https://sedac.ciesin.columbia.edu (accessed on 1 March 2022)). The linear features (road and water) were downloaded from DivaGis (www.diva.gis.org (accessed on 1 March 2022)). Variables were resampled with the resolution of 30 arcsecs and ~1 km^2^ spatial resolution using the spatial-analyst tool in ArcGIS 10.9. The Pearson correlation test was performed to identify and address significant collinearity among variables. Variables with a Pearson coefficient greater than 0.8 (rs > 0.8) were dropped from further analysis [39]. Finally, 18 environmental variables we assumed might have an ecological effect on the distribution of the species were retained for modelling the habitat suitability of musk deer (Supplementary Table S1).

#### 2.5.3. Model Building

The SDMs are commonly used to model species–environment relationships and forecast species spatial distributions [26,40,41,42,43,44]. The procedures and methods for performing SDM have also evolved to produce more reliable habitat suitability maps [45,46,47,48]. The present study implemented an ensemble modelling approach, using the r package ‘biomod2′ [48], to model the current distribution of musk deer in the Western Himalayas. We used eight modelling algorithms for developing the model, viz., generalised boosting modelling (GBM), Random Forest (RF), generalised linear modelling (GLM), artificial neural network (ANN), flexible discriminate analysis (FDA), maximum entropy (MAXENT), multivariate adaptive regression splines (MARS), and generalised additive models (GAM). The data were split into 80% and 20% for training and testing, respectively, with five repetitions. Furthermore, to enhance the accuracy of simulated distribution and to minimise spatial deviation, we randomly selected 1000 pseudoabsence points. This process was repeated twice during the model construction phase. The model performance was evaluated using the model evaluation score’s Receivers Operating Curve (ROC) and True Skill Statistic (TSS). After generating all model outputs, the best-performing models were used to develop the ensemble model, a robust strategy widely used to investigate species distribution [49,50,51]. The variable importance scores were extracted from the models using in-built ‘biomod2′ functions. The final model was selected based on the TSS threshold for the ensemble model [52].

## 3. Result

### 3.1. Camera Trapping and Non-Invasive DNA Sampling

**Figure 2:**
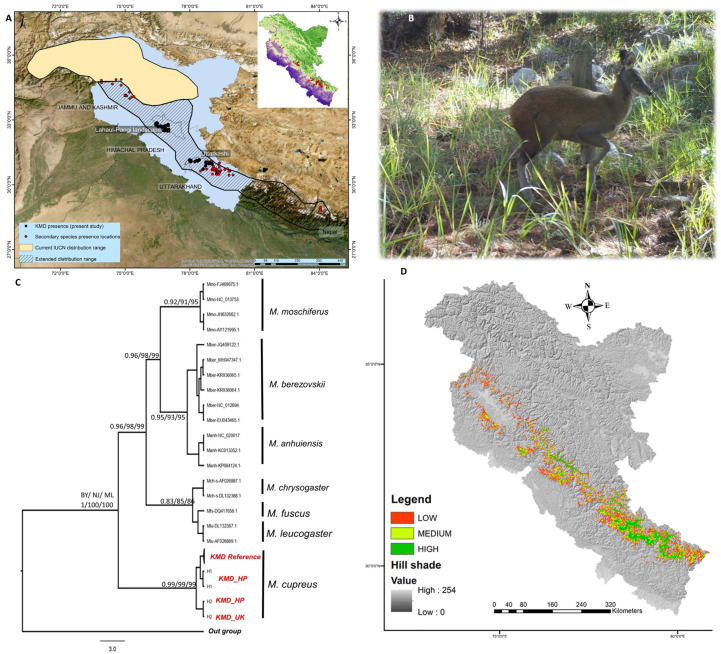
Kashmir musk deer presence: (**A**) map showing district highlighted with first confirmed records of KMD in the Lahaul–Pangi landscape, Himachal Pradesh, and Uttarkashi, Uttarakhand, Western Himalayas, with current IUCN range and proposed extended range of KMD based on the current study and the published literature (Kumar et al. [19] and Singh et al. [7,20]; (**B**) camera trap image of KMD in Gangotri National Park, Uttarkashi; (**C**) phylogenetic tree showing the relationship of KMD with other musk deer species using mitochondrial Cytochrome b gene, and values represented on nodes are bootstraps support and posterior probability: BY—Bayesian, NJ—Neighbor Joining, and ML—maximum likelihood; (**D**) predicted suitable range of KMD in the Western Himalayas.

Based on the combined effort of 255 camera traps deployed in different habitats of the musk deer range in the Lahaul–Pangi landscape and Uttarkashi, a total of 103 independent captures of musk deer were recorded (Figure 2A,B). All the captured images of musk deer were identified as Kashmir musk deer (KMD; *Moschus cupreus*). The capture rate was 0.006 ± 0.002 in Uttarkashi and 0.03 ± 0.008 in the Lahaul Valley. Among the 220 (106 Lahaul, 99 Uttarakashi, and 15 Pangi Valley) trails traversed, we identified 43 latrine sites of KMD in Uttarkashi (11) and the Lahaul–Pangi (32) landscape. The mean encounter rate of KMD was 0.26 ± 0.07 in the Lahaul Valley and 0.012 ± 0.007 in Uttarkashi.

A total of 37 sequences (19 from the Lahaul Valley and 18 from Uttarkashi) were successfully generated. All sequences were identified as Kashmir musk deer with >98% similarity. Hence, the generated DNA sequences from the processed faecal samples from Himachal Pradesh and Uttarakhand confirmed the presence of KMD in the Uttarkashi and Lahaul–Pangi landscapes; these also included two sequences of KMD from NCBI. All 39 (37 from the present study and 2 NCBI) sequences were grouped into three haplotypes with nucleotide diversity of 0.00356 and haplotype diversity of 0.555 (Table S2). We observed an intra-species K2P sequence divergence of 0.00–0.009 and an inter-species sequence divergence of 0.020–0.115 (Table S3). In the phylogenetic tree, our sequences clustered with the sequences of KMD and formed a separate basal clade when compared with other species supported with high bootstrap values (Figure 2C). Our results, for the first time, confirmed the presence of KMD in Himachal Pradesh and Uttarkashi (Uttarakhand), Western Himalayas, using DNA-based tools supported with photographic evidence through camera traps.

### 3.2. Species Distribution Modelling

**Figure 3:**
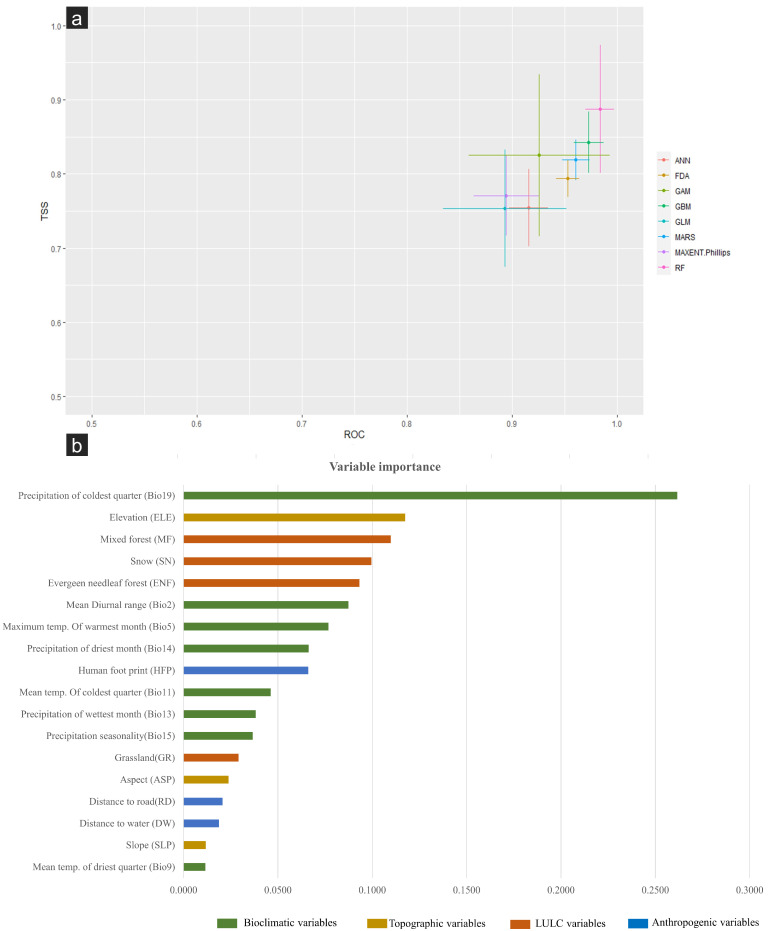
Variable importance: (**a**) model selection by TSS threshold value above 0.8 for Kashmir musk deer distribution model in the Western Himalayas; (**b**) graph representing the variable importance of different predictors used in predicting the distribution of Kashmir musk deer in the Western Himalayas; (i) bioclimatic predictors: Bio2, Bio5, Bio9, Bio11, Bio13, Bio14, Bio15, and Bio19, (ii) LULC predictors: MF, SN, ENF, and GD, (iii) topographic predictors: ELE, SLP, and ASP, and (iv) anthropogenic predictors: DR, DW, and HFP.

This study employed the ensemble modelling approach for predicting the suitable habitat of KMD in the Western Himalayas. Out of the eight modelling algorithms used for building ensemble models, the best-performing models were RF, MARS, GBM, and GAM. Model selection was based on the TSS value being greater than 0.8 (Figure 3a). KMDs are predicted to inhabit a narrow belt of high altitudes in the Western Himalayas. The present study revealed that out of the total 324,666 km^2^ area of the study landscape, only 20,690 km^2^ (6.97%) are identified as suitable habitat for KMD in the Western Himalayas, which are spread between 2500 and 4500 m. Further, out of the total suitable habitat (20,690 km^2^), about 9921 km^2^ is low-suitable, 6118 km^2^ is medium-suitable, and 4650 km^2^ is classified as highly suitable for KMD (Figure 2D). Among all 18 variables used in modelling, the precipitation of the coldest quarter (bio19) and elevation were the top influential factors governing the distribution of KMD in the Western Himalayas, with variable importance of 0.22 and 0.17, respectively (Figure 3b). The variables associated with land cover type and distance from mixed forests contributed the most to determine the distribution of KMD.

## 4. Discussion

For the first time, we documented the presence of KMD in the Lahaul Valley, Himachal Pradesh, and Uttarkashi, Uttarakhand. The suitable habitat of KMD, identified with the species distribution model, is mainly confined to high-elevation, wet areas. Our results, thus, support the findings of the previous studies that reported KMD in the Western Himalayas without solid evidence. However, the other two species previously reported in the region, i.e., Himalayan musk deer and Alpine musk deer, were surprisingly not observed during the present study. Hence, we suggest that the presence of Himalayan musk deer and Alpine musk deer was wrongly reported in the Western Himalayas, especially in Uttarakhand and Himachal Pradesh. Our results are consistent with those of Singh et al. [7,20], who found that KMD extends into central Nepal [20] but did not report records of Alpine musk deer in Nepal. Recent images and video captured in June 2021 from Chamoli District by the Uttarakhand Forest Department, after ten years, were also identified as KMD (media report, 2021). Further, the phylogenetic tree shows that our sequences clustered with the sequences of Kashmir musk deer and formed a separate and basal clade from other species of musk deer. The KMD clade is highly divergent, possibly due to geological and environmental changes that had a prominent impact on the species evolution, including musk deer in the Himalayas and the Tibetan Plateau [3]. Considering the distribution of KMD in the Western Himalayas’ eco-climatic zone [53,54], which is geographically separated, its congeneric species are distributed eastwards (Eastern Himalayas) and experience variability in vegetation, temperature, precipitation, and topographic barrier [53] that may result in observed paraphyletic clades (Figure 2C). Thus, the conservation, distribution, and status of KMD need to be extended throughout the Western Himalayas and up to central Nepal.

Our study suggests that KMD inhabit a narrow range of the Western Himalayas between 2500 and 4500 m [7], corresponding to only 6.9% of the Western Himalayas; the rest of the region is likely a non-suitable habitat for the species. The precipitation of the coldest quarter (bio19) and elevation were the top contributing factors positively influencing the distribution of KMD in the Western Himalayas. The positive association with precipitation likely results from vegetation because the growth-limiting factor for vegetation is the moisture available during the pre-monsoon season, which plays a vital role in governing the distribution of KMD [7]. Moreover, the predicted KMD distribution range between 2500 and 4500 m indicates that KMD prefers to live at higher elevations and is sensitive to anthropogenic disturbances. Hence, the positive association with elevation may be related to the avoidance of human disturbances by the species in the landscape, which corroborates with the findings of previous studies [15,55,56,57]. We found that most of the habitat patches in the Uttarkashi and Lahaul–Pangi landscapes are restricted to a narrow range threatened by anthropogenic pressures such as intensive fuel-wood cutting, livestock grazing, and fodder collection. Further, this species is also heavily poached for its musk pods, leading to the rapid decline of its population [9,16]; hence strict enforcement must be implemented to reduce this pressure. While interacting with locals and during sampling, we also observed that poaching for musk pods and encroachment are the two major threats responsible for the declining population of musk deer. Instead of lamenting past follies, adaptive conservation planning is required to conserve the musk deer inside and outside protected areas. Preserving the existence of this rare animal is critical at present, as it is rapidly approaching regional extinction due to numerous associated threats.

Although we used combined approaches of DNA-based identification and camera trapping to collect the evidences of KMD in the study sites, our study has some limitations. The samples may not represent the entire distribution range of Kashmir musk deer in the Western Himalayas. Thus, further intensive sampling is required to gain better insights into the distribution range of KMD, understand the genetic diversity and demographic history, and cover taxonomic diversity. Nonetheless, the present study provides valuable distribution information using multiple methods. It paves the way for using these methods in population estimation, habitat ecology, and assessing suitable habitats for KMD and other elusive species.

## 5. Conclusions

This study is the first to confirm the presence of Kashmir musk deer from Uttarkashi District of Uttarakhand and the Lahaul–Pangi landscape, Himachal Pradesh, using camera trapping and DNA-based approaches. Although previous studies reported the presence of Alpine musk deer and Himalayan musk deer, we found that only Kashmir musk deer occur in Western Himalaya. Our study also predicts the suitable habitat of KMD in the Western Himalayas and suggests that precipitation and elevation are the most influential factors governing the distribution of KMD. The KDM is vulnerable to climate change because its habitat is located in the Himalayas high-elevation zones. These areas are particularly susceptible to global warming. In addition, the relationship between precipitation, temperature and the species’ habitat further amplifies this vulnerability. Therefore, KMD is at risk from climate change due to its habitat sensitivity and reliance on specific precipitation and temperature conditions. Thus, the identified suitable habitats inside and outside the protected areas should be prioritised for conservation and management planning. This is particularly important in lieu of the fact that the Lahaul Valley does not have any protected areas, which is a serious issue for the long-term survival of this species. Hence, we urge the creation of a protected area based on the identified suitable habitat of KMD, which is also home to some other conservation priority species, such as the snow leopard in the Lahaul Valley. Although KMD is a top conservation priority species of the Himalayan region, no long-term, robust data is available on its population trends or habitat ecology. Since species populations appear to be declining at an alarming rate, additional data on the species’ habitat ecology and behaviour are necessary. Therefore, we recommend long-term studies on the population and habitat ecology of KMD by using the baseline information generated in the present study for long-term conservation.

## Data Availability

Data can be made available upon request.

## Supplementary Materials

The following supporting information can be downloaded at https://www.mdpi.com/article/10.3390/biology12060786/s1. Table S1: Environmental variables used for modelling the distribution of KMD in the Western Himalayas; Table S2: Mitochondrial genetic diversity in Kashmir Musk Deer; Table S3: Sequence divergence between the species where shaded rows and columns presenting sequence divergence in Kashmir Musk Deer.

## References

[1] Groves C.P., Wang Y., Grubb P. (1995). Taxonomy of musk deer, genus *Moschus* (Moschidae, Mammllia). Acta Theriol. Sin..

[2] Ostrowski S., Rahmani H., Ali J.M., Ali R., Zahler P. (2016). Musk deer *Moschus cupreus* persist in the eastern forests of Afghanistan. Oryx.

[3] Pan T., Wang H., Hu C., Sun Z., Zhu X., Meng T. (2015). Species Delimitation in the Genus *Moschus* (Ruminantia: Moschidae) and Its High-Plateau Origin. PLoS ONE.

[4] Shukla M., Joshi B.D., Kumar V.P., Thakur M., Mehta A.K., Sathyakumar S., Goyal S.P. (2018). Species dilemma of musk deer (*Moschus* spp.) in India: Molecular data on cytochrome c oxidase I suggests distinct genetic lineage in Uttarakhand compared to other *Moschus* species. Anim. Biotechnol. Bull..

[5] Zaitsev V.A. (2006). Musk Deer: Ecology, Population Dynamics, Conservation Prospects.

[6] Sathyakumar S., Rawat G., Johnsingh A.J.T., Johnsingh A.J.T., Manjrekar N. (2015). Order Artiodactyla Family Moschidae Evolution, Taxonomy and Distribution. Mammals of South Asias.

[7] Singh P.B., Mainali K., Jiang Z., Thapa A., Subedi N., Awan M.N., Ilyas O., Luitel H., Zhou Z., Hu H. (2020). Projected distribution and climate refugia of endangered Kashmir musk deer *Moschus cupreus* in greater Himalaya, South Asia. Sci. Rep..

[8] IUCN (2018). The IUCN Red List of Threatened Species. http://www.iucnredlist.org/.

[9] Khan A.A., Qureshi B.U.D., Awan M.S. (2006). Impact of musk trade on the decline in Himalayan musk deer *Moschus chrysogaster* population in Neelum Valley, Pakistan. Curr. Sci..

[10] Wangdi T., Tobgay S., Dorjee K., Dorji K., Wangyel S. (2019). The distribution status and conservation of the Himalayan Musk Deer *Moschus chrysogaster* in Sakteng Wildlife Sanctuary. Glob. Ecol. Conserv..

[11] Yang Q., Meng X., Xia L., Feng Z. (2003). Conservation status and causes of decline of musk deer (*Moschus* spp.) in China. Biol. Conserv..

[12] Green M.J.B. (1986). The distribution, status and conservation of the Himalayan musk deer *Moschus chrysogaster*. Biol. Conserv..

[13] Timmins R.J., Duckworth J.W. *Moschus cupreus*. The IUCN Red List of Threatened Species. 2015, e.T13901A61977764. http://www.iucnredlist.org/details/13901/0.

[14] CITES (2015). Official Documents Appendices I, II and III. Convention on International Trade in Endangered Species of Wild Fauna and Flora. www.cites.org/eng/append/latest_appendices.shtml.

[15] Ilyas O. (2014). Status habitat use and conservation of Alpine musk deer (*Moschus chrysogaster*) in Uttarakhand Himalayas. J. Appl. Anim. Res..

[16] IUCN The IUCN Red List of Threatened Species. 2023, Version 2022-2. 2307-8235. https://www.iucnredlist.org.

[17] Pal R., Bhattacharya T., Qureshi Q., Buckland S.T., Sathyakumar S. (2021). Using distance sampling with camera traps to estimate the density of group-living and solitary mountain ungulates. Oryx.

[18] Ali M. (2014). Habitat suitability modelling for exploration of the spatial distribution of Kashmir musk deer in Dachigam National Park. Am. J. Environ. Sci..

[19] Kumar A., Singh B., Sahoo S., Gautam K.B., Gupta S.K. (2022). Genetic evidence indicates new distribution record of endangered Kashmir musk deer (*Moschus cupreus*) with range expansion in Uttarakhand, India. Oryx.

[20] Singh P.B., Khatiwada J.R., Saud P., Jiang Z. (2019). mtDNA analysis confirms the endangered Kashmir musk deer extends its range to Nepal. Sci. Rep..

[21] Bowkett A.E., Rovero F., Marshall A.R. (2008). The use of camera-trap data to model habitat use by antelope species in the Udzungwa Mountain forests, Tanzania. Afr. J. Ecol..

[22] Rovero F., Martin E., Rosa M., Ahumada J.A., Spitale D. (2014). Estimating Species Richness and Modelling Habitat Preferences of Tropical Forest Mammals from Camera Trap Data. PLoS ONE.

[23] Albayrak T., Giannatos G., Kabasakal B. (2012). Carnivore and Ungulate Populations in the Beydaglari Mountains (Antalya, Turkey): Border Region between Asia and Europe. Pol. J. Ecol..

[24] Joshi B.D., Singh S.K., Singh V.K., Jabin G., Ghosh A., Dalui S., Singh A., Priyambada P., Dolker S., Mukherjee T. (2022). From poops to planning: A broad non-invasive genetic survey of large mammals from the Indian Himalayan Region. Sci. Total Environ..

[25] Kaya S., Kabasakal B., Erdoğan A. (2023). Geographic Genetic Structure of Alectoris chukar in Türkiye: Post-LGM-Induced Hybridization and Human-Mediated Contaminations. Biology.

[26] Guisan A., Thuiller W. (2005). Predicting species distribution: Offering more than simple habitat models. Ecol. Lett..

[27] Sharief A., Singh H., Dutta R., Kumar V., Bhattacharjee S., Mukherjee T., Joshi B.D., Ramesh C., Thakur M., Sharma L.K. (2023). Estimating Occupancy and Abundance of Endangered Kashmir Musk Deer (*Moschus cupreus*) in Uttarkashi, Uttarakhand. Ind. J. Ecol..

[28] Pelletier T.A., Crisafulli C., Wagner S., Zellmer A.J., Carstens B.C. (2015). Historical Species Distribution Models Predict Species Limits in Western *Plethodon* Salamanders. Syst Biol..

[29] Verma S.K., Singh L. (2003). Novel universal primers establish identity of an enormous number of animal species for forensic application. Mol. Ecol. Notes.

[30] Hall T.A. (1999). BioEdit: A user-friendly biological sequence alignment editor and analysis program for Windows 95/98/NT. Nucleic Acids Symp. Ser..

[31] Tamura K., Stecher G., Kumar S. (2021). MEGA11: Molecular evolutionary genetics analysis version 11. Mol. Biol. Evol..

[32] Drummond A.J., Rambaut A. (2007). BEAST: Bayesian evolutionary analysis by sampling trees. BMC Evol. Biol..

[33] Nylander J.A.A. (2004). Mr Modeltest v2. Program Distributed by the Author Evolutionary Biology Centre.

[34] Menon V. (2018). Mammals of India: A Natural History.

[35] Joshi B.D., Sharief A., Kumar V., Kumar M., Dutta R., Devi R., Singh A., Thakur M., Sharma L.K., Chandra K. (2020). Field Testing of Different Methods for Monitoring Mammals in Trans-Himalayas: A Case Study from Lahaul and Spiti. Glob. Ecol. Conserv..

[36] Tobler M.W., Carrillo-Percastegui S.E., Leite Pitman R., Mares R., Powell G. (2008). An evaluation of camera traps for inventorying large-and medium-sized terrestrial rainforest mammals. Anim. Conserv..

[37] Nandy S., Neethu Lakshmi M., Kushwaha S.P.S. (2020). Habitat Suitability Analysis of Himalayan Musk Deer (*Moschus leucogaster*) in Part of Western Himalaya, India. J. Indian Soc. Remote Sens..

[38] Hijmans R.J., Cameron S.E., Parra J.L., Jones P.G., Jarvis A. (2005). Very high resolution interpolated climate surfaces for global land areas. Int. J. Climatol..

[39] Warren D.L., Glor R.E., Turelli M. (2010). ENMTools: A toolbox for comparative studies of environmental niche models. Ecography.

[40] Elith J., Graham C.H., Anderson R.P., Dudik M., Ferrier S. (2006). Novel methods improve prediction of species’ distributions from occurrence data. Ecography.

[41] Guisan A., Zimmermann N.E. (2000). Predictive habitat distribution models in ecology. Ecol. Model..

[42] Hijmans R.J., Elith J. (2013). Species Distribution Modeling with R.

[43] Dutta R., Mukherjee T., Sharief A., Singh H., Kumar V., Joshi B.D., Banerjee D., Thakur M., Sharma L.K. (2022). Climate change may plunder the facultative top predator Yellow-throated Martin from the Hindu-Kush Himalayan Region. Ecol. Inform..

[44] Araújo M.B., New M. (2007). Ensemble forecasting of species distributions. Trends Ecol. Evol..

[45] Buisson L., Thuiller W., Casajus N., Lek S., Grenoulliet G. (2010). Uncertainty in ensemble forecasting of species distribution. Glob. Chang. Biol..

[46] Kindt R. (2018). Ensemble species distribution modelling with transformed suitability values. Environ. Model. Softw..

[47] Thuiller W., Lafourcade B., Engler R., Araújo M.B. (2009). BIOMOD—A platform for ensemble forecasting of species distributions. Ecography.

[48] Thuiller W., Georges D., Engler R., Breiner F., Georges M.D., Thuiller C.W. (2016). Package ‘Biomod2’. Ensemble Platform for Species Distribution Modelling.

[49] Elith J., Leathwick J.R. (2009). Species distribution models: Ecological explanation and prediction across space and time. Annu. Rev. Ecol. Evol. Syst..

[50] Miller J. (2010). Species distribution modelling. Geogr. Compass..

[51] Woodman S.M., Forney K.A., Becker E.A., DeAngelis M.L., Hazen E.L., Palacios D.M., Redfern J.V. (2019). Esdm: A tool for creating and exploring ensembles of predictions from species distribution and abundance models. Methods Ecol. Evol..

[52] Allouche O., Tsoar A., Kadmon R. (2006). Assessing the accuracy of species distribution models: Prevalence, kappa and the true skill statistic (TSS). J. Appl. Ecol..

[53] Valdiya K.S. (2002). Emergence and evolution of Himalaya: Reconstructing history in the light of recent studies. Prog. Phys. Geogr..

[54] Pandit M.K. (2017). Life in the Himalaya: An Ecosystem at Risk.

[55] Sathyakumar S. (1994). Habitat Ecology of Major Ungulates in Kedarnath Musk Deer Sanctuary, Western Himalaya. Ph.D. Thesis.

[56] Subedi A., Aryal A., Koirala R.K., Timilsina Y.P., Meng X. (2012). Habitat ecology of Himalayan Himalayan Musk Deer (*Moschus chrysogaster*) in Manaslu Conservation Area, Nepal. Int. J. Zool Res..

[57] Vinod T.R., Sathyakumar S. (1999). Ecology and conservation of mountain ungulates in Great Himalayan National Park, Western Himalaya. An Ecological Study of the Conservation of Biodiversity and Biotic Pressures in the Great Himalayan National Park Conservation Area—An Ecodevelopment Approach.

