# Supplementary material for: Empirical Data Suggest That the Kashmir Musk Deer (Moschus cupreus, Grubb 1982) Is the One Musk Deer Distributed in the Western Himalayas: An Integration of Ecology, Genetics and Geospatial Modelling Approaches
Source: Biology (Basel). 2023 May 29;12(6):786. doi: 10.3390/biology12060786 (PMC10295256; doi:10.3390/biology12060786)
Supplement: Supplementary file 1 [file biology-12-00786-s001.zip › biology-2381070-supplementary.pdf]

Supplementary File

Table S1. Environmental variables used for modelling the distribution of KMD in the western Himalayas.

S. No.	Variable	Code	Data	Source
1	Bio2	Bio2	Worldclim	Worldclim
2	Bio5	Bio5		
3	Bio9	Bio9		
4	Bio11	Bio11		
5	Bio13	Bio13		
6	Bio14	Bio14		
7	Bio15	Bio15		
8	Bio19	Bio19		
9	Grass land (dominated by herbaceous annuals)	Grass	MCD12Q1	USGS
10	Mixed forest	MF		
11	Evergreen needle leaf forest	ENF		
12	Permanent snow and ice (at least 60% of area is covered by snow for at least 10 months of the year)	SN		
13	Distance to water	WA	Calculated using log Euclidean distance (Arcgis 10)	Diva gis
14	Distance to road	RD		
15	Elevation	ELE	SRTM	USGS
16	Slope	SLP		
17	Aspect	ASP		
18	Human footprint	HFP	EARTHDATA	SEDAC

Table S2. Mitochondrial genetic diversity in Kashmir Musk deer.

	N	P	H	Nucleotide Diversity(π)	Hd
KMD	39	3	3	0.00356±0.0001	0.555±0.037

Table S3. Sequence divergence between the species and rows and columns shaded are shown sequence divergence in Kashmir Musk deer.

	KMD_H1	KMD_H2	KMD_H3	Mfus	Mlu	Mch	Man	Mber	Mmo
KMD_H1									
KMD_H2	0.007								
KMD_H3	0.003	0.009							
Mfus	0.114	0.114	0.118						
Mlu	0.107	0.107	0.111	0.010					
Mch	0.111	0.111	0.115	0.020	0.016				
Man	0.103	0.103	0.107	0.086	0.073	0.076			
Mber	0.092	0.092	0.095	0.075	0.062	0.066	0.023		
Mmo	0.107	0.107	0.111	0.094	0.084	0.096	0.073	0.069	

Siberian musk deer *(Moschus moschiferus)*, Chinese forest musk deer (*M. berezovskii*), Anhui musk deer (*M. anhuiensis)*, Alpine musk deer (*M. chrysogaster*), Himalayan musk deer (*M. leucogaster)*, Mfus- (*M. fuscus*) and Kashmir musk deer (*M. cupreus*).
